# Novel excitation-contraction coupling related genes reveal aspects of muscle weakness beyond atrophy—new hopes for treatment of musculoskeletal diseases

**DOI:** 10.3389/fphys.2014.00037

**Published:** 2014-02-18

**Authors:** Heather Manring, Eduardo Abreu, Leticia Brotto, Noah Weisleder, Marco Brotto

**Affiliations:** ^1^Department of Physiology and Cell Biology, Davis Heart and Lung Research Institute, The Ohio State University Wexner Medical CenterColumbus, OH, USA; ^2^Muscle Biology Research Group, School of Nursing and Health Studies, University of Missouri-Kansas CityKansas City, MO, USA; ^3^Basic Medical Sciences Pharmacology, School of Medicine, University of Missouri-Kansas CityKansas City, MO, USA; ^4^Basic Medical Sciences Pharmacology, School of Pharmacy, University of Missouri-Kansas CityKansas City, MO, USA

**Keywords:** musculoskeletal diseases, MG29, MTMR14, sarcalumenin, KLF15, calcium homeostasis, sarcopenia, aging

## Abstract

Research over the last decade strengthened the understanding that skeletal muscles are not only the major tissue in the body from a volume point of view but also function as a master regulator contributing to optimal organismal health. These new contributions to the available body of knowledge triggered great interest in the roles of skeletal muscle beyond contraction. The World Health Organization, through its Global Burden of Disease (GBD) report, recently raised further awareness about the key importance of skeletal muscles as the GDB reported musculoskeletal (MSK) diseases have become the second greatest cause of disability, with more than 1.7 billion people in the globe affected by a diversity of MSK conditions. Besides their role in MSK disorders, skeletal muscles are also seen as principal metabolic organs with essential contributions to metabolic disorders, especially those linked to physical inactivity. In this review, we have focused on the unique function of new genes/proteins (i.e., MTMR14, MG29, sarcalumenin, KLF15) that during the last few years have helped provide novel insights about muscle function in health and disease, muscle fatigue, muscle metabolism, and muscle aging. Next, we provide an in depth discussion of how these genes/proteins converge into a common function of acting as regulators of intracellular calcium homeostasis. A clear link between dysfunctional calcium homeostasis is established and the special role of store-operated calcium entry is analyzed. The new knowledge that has been generated by the understanding of the roles of previously unknown modulatory genes of the skeletal muscle excitation-contraction coupling (ECC) process brings exciting new possibilities for treatment of MSK diseases, muscle regeneration, and skeletal muscle tissue engineering. The next decade of skeletal muscle and MSK research is bound to bring to fruition applied knowledge that will hopefully offset the current heavy and sad burden of MSK diseases on the planet.

## Introduction to the global problem

The Global Burden of Disease Study (GBD) of 2010 estimates 1.7 billion people worldwide are affected by musculoskeletal disorders (MSDs). Among the almost 300 diseases and injuries evaluated in the GBD study of 2010, MSDs rank as the second greatest cause of disability according to the calculated years lived with disability (YLDs) for affected individuals. This equates to roughly 21.3% of all YLDs. MSDs only rank below mental and behavioral disorders with respect to this measure. Between 1990 and 2010, a 44.7% increase in the YLDs of musculoskeletal disorders was observed (Vos et al., 2010). When disorders are evaluated in terms of disability-adjusted life years (DALYs), MSDs ranked fourth below cardiovascular diseases, neoplasms, and mental disorders. DALYs give a more accurate representation of the drivers of poor health by accounting for both disability and death associated with a disorder rather than basing the impact solely on the number of deaths over time. In 2010, MSDs accounted for roughly 6.8% of total DALYs globally which increased from the estimated 4.7% in 1990 (Murray et al., 2010). The distribution and impact of MSDs is relatively equal globally as these conditions are not considered indigenous to a specific region. The GBD of 2010 suggests that healthcare systems need to focus on developing a policy to deal with the increasing burden caused by MSDs (Murray et al., 2010; Vos et al., 2010).

Musculoskeletal disorders include a variety of conditions that affect muscles, bones, and joints throughout the body. The impact of MSDs on daily life ranges from minimal discomfort to debilitating pain that considerably affects the performance of simple everyday activities. In terms of severity, MSDs encompass a broad spectrum of symptoms ranging from minor conditions to major disorders, including arthritis, back and neck pain, and muscle wasting disorders. In this review we will focus on some of the most severe of these disorders, specifically skeletal muscle wasting disorders that have the broadest impact on human health and patient outcomes. The increased prevalence of muscle wasting disorders appears to be in part due to the increasing life span of humans with age as a contributing factor in approximately one-third of documented MSDs (561 million people). A 49.9% increase in DALYs between 1990 and 2010 was observed for all types of muscle wasting disorders, which is slightly larger than the change observed for MSDs in general (Murray et al., 2010). Muscle wasting is a comorbidity of the ever increasing conditions of heart failure and cancer in addition to its association with skeletal muscle disorders. With its rise in prevalence, muscle wasting disorders and their underlying mechanisms are of great importance in an effort to provide appropriate treatments (Teixeira Vde et al., 2012). While multiple MSDs contribute to changes in human health, skeletal muscle wasting will be the major focus of this review.

## Muscle wasting disorders

### Muscle wasting is much more than muscle loss

In skeletal muscle, the number of cells (muscle fibers) present in an anatomical muscle stabilizes early in life and remains constant into adulthood, after which time increased muscle mass and strength is dependent on an increase in the size of muscle fibers (hypertrophy). Injured muscle fibers can be repaired or replaced by activation of neighboring satellite cells that will proliferate and repair damaged muscle fibers. A decrease in muscle fiber size is a factor contributing to muscle weakness. While muscle weakness is a shared characteristic of many skeletal muscle wasting disorders including atrophy, sarcopenia, myopathy, and others, the long term outcome of these disorders in addition to the biochemical and molecular processes driving them can be distinct (Romanick et al., 2013). For this reason, further research on the mechanisms behind each disorder and evaluation of possible therapeutic interventions for the associated muscle weakness is of vital importance.

Atrophy is the loss of myofiber size and quantity most commonly due to disuse of muscles (Lexell, 1993; Brooks and Faulkner, 1994; Schakman et al., 2013). While the pathophysiological effects of atrophy caused by inactivity can typically be restored by physical activity, these effects are not easily reversed in situations such as sarcopenia and other muscle wasting disorders (Faulkner et al., 1995; Bortz and Bortz, 1996; Gonzalez et al., 2000; Zahn et al., 2006; Romero-Suarez et al., 2010). In these conditions, recovery of muscle strength caused by the functional and physical loss of muscle fibers is very difficult. This continued and irreversible detrimental effect on muscle fibers leads to fragility and eventually hinders the quality of life and independence of individuals. Sarcopenia has been specifically referred to as muscle atrophy and wasting that accompanies aging. A variety of molecular targets and processes proposed to be involved in sarcopenia include muscle proteolysis, increased cellular autophagy, aberrant activation of Ca^2+^-activated proteases and proteasomes, and dysfunction or loss of satellite cells (Teixeira Vde et al., 2012; Romanick et al., 2013). While sarcopenia is considered a normal part of healthy aging, studies suggest that the progression of sarcopenia can be slowed if the specific molecular process responsible for its pathophysiological effects can be determined and specifically targeted (Romanick et al., 2013). In fact, the complexity of these diseases associated with muscle wasting is further compounded by an intriguing signature where muscle mass does not necessarily match functional status (i.e. muscle contractile force and muscle power) (Lowe et al., 2002, 2004; Romero-Suarez et al., 2010; Manini and Clark, 2012; Russ et al., 2012). Multiple factors contribute to this phenomenon, including increased fatty infiltration and an increased prevalence of myosin type I expression in aging skeletal muscle. While the loss of muscle mass itself is detrimental to health because of its obvious metabolic consequences, the loss of functional capacity surpasses the muscle content loss and aggravates these conditions (Lowe et al., 2002, 2004; Mitchell et al., 2012; Romanick et al., 2013).

It is important to distinguish between muscle wasting and muscle weakness that can be two distinct processes involving loss of muscle mass or loss of functional muscle output respectively. Muscle weakness itself is a major contributor to morbidity, risk of falls, and mortality (Brotto and Abreu, 2012). Our research groups have proposed since the early 2000's that the definition of sarcopenia for example must not be limited to “loss of muscle mass.” It is still puzzling that despite many recent efforts sarcopenia is not defined as a disease; an International Disease Code (IDC) still does not exist for sarcopenia, which should be defined as a disease of aging characterized by a loss of muscle strength that surpasses the loss of muscle mass. Muscle fatigue is another essential characteristic of muscle that is commonly affected in muscle wasting disorders and in the aging process. In general, muscle fatigue is defined as a reversible decline in the ability of a muscle to create force either due to repetitive or continued activity. Muscle fatigue is thought to be a biological process to minimize damage to muscles that is produced by overexertion; however, the exact cellular mechanisms contributing to muscle fatigue remain to be fully clarified (Bruton et al., 1998; Nagaraj et al., 2000; de Paula Brotto et al., 2001; Brotto et al., 2002). A better understanding of muscle weakness, atrophy and fatigue are necessary to be able to develop medical interventions to restore muscle strength and reduce the fatigability of muscles, which are essential for effective treatment of muscle wasting disorders (Romanick et al., 2013).

## Muscle myopathies

### A general definition

Myopathies are a distinct group of muscle wasting disorders. Myopathies are neuromuscular disorders in which the main symptom is muscle weakness caused by dysfunction of muscle fibers (Chawla, 2011). There are many types of myopathies including inherited myopathies such as muscular dystrophies and acquired myopathies (Sewry, 2008). The distinct biochemical and cellular mechanisms underlying the pathology of these myopathies lead to greatly varied prognoses and treatments. Only palliative care is currently available for many of these disorders due to the limited understanding of their pathology.

### The special case of ethanol-induced myopathy

Ethanol-induced myopathy is acquired following excessive and/or chronic consumption of alcohol and is roughly five times more common than alcoholic cirrhosis (Estruch et al., 1993). Multiple studies have confirmed the prevalence of this myopathy and determined that loss of muscle bulk as well as weakness is the result. Abstinence and nutritional support facilitates recovery of these patients but muscle strength does not revert to baseline values suggesting this is an irreversible process (Urbano-Marquez and Fernandez-Sola, 2004). While the causative agent of this myopathy has been known, the steps leading to its development are unknown. Over the past 30 years progress has been made in understanding of this disease entity. The mechanisms underlying this multi-factorial disease include disruption of protein metabolism, signal transduction, and improper gene regulation (Urbano-Marquez and Fernandez-Sola, 2004; Noordzij et al., 2007; Gonzalez-Reimers et al., 2010; Chawla, 2011). *In vivo* and *in vitro* studies using models for alcohol-induced myopathy observed a decrease in muscle mass specifically fast twitch (Type II) fibers with multiple possible contributory mechanisms identified. Protein synthesis of myofibrillar proteins is impaired at the initiation step of translation by alcohol (Preedy and Peters, 1988; Urbano-Marquez et al., 1995; Lang et al., 1999). Analysis of mRNA and protein expression following chronic and acute alcohol exposure determined multiple pathways and processes are modified by alcohol including the ATP-dependent multi-catalytic proteasome pathway. In skeletal muscles, alcohol induced apoptosis and changed expression and activity of the mTOR pathway (Lang et al., 2003; Nakahara et al., 2003, 2006; Hong-Brown et al., 2006). An additional contributory mechanism being evaluated in this disease is oxidative damage due to increased reactive oxygen species or changes in prevalence of antioxidants (Hofer et al., 2005; Fernandez-Sola et al., 2007). Recently, corollary studies by Preedy et al. have firmly established that ethanol-induced myopathies could account for more than 50% of all cases of myopathies (Preedy et al., 2001a,b). Despite the prevalence of this syndrome, effective treatments to either prevent or to cure this condition remain unavailable.

### Muscular dystrophy

Muscular dystrophies encompass another group of degenerative myopathies which involve progressive muscle weakness that often presents at birth or starting in early childhood However, there are many types of muscular dystrophy that vary in their underlying genetic foundation, the severity of disease including the body regions affected, the time of onset, and the rate of disease progression. These distinct types are associated with perturbations of various genes including *DYSF* (dysferlin), *DUX4* (double homeobox 4), *LMN* (Lamin), and the *DMD* gene that encodes the dystrophin protein (Rahimov and Kunkel, 2013). Disruption of these genes and several others lead to muscle damage partially attributed to defects in sarcolemmal membrane stability and repair. This damage is associated with progressive muscle weakness, which appears to have a greater functional consequence than loss of muscle mass.

Duchenne muscular dystrophy (DMD) is a recessive X-linked form of muscular dystrophy that affects approximately 1 in 3600 boys. Symptoms of DMD tend to appear at or before six years of age with muscle weakness in the legs and pelvis followed by other body regions as the disorder progresses. Muscle deterioration and immobility eventually lead to paralysis with an average lifespan of around 25 years. The observed muscle degeneration is associated with mutations in the dystrophin gene. Dystrophin is an important structural component of the dystroglycan complex of the cell membrane that contributes to maintaining muscle fiber strength, preventing muscle injury, and retaining the mechanical stability of muscle cells (Rahimov and Kunkel, 2013). Absence of functional dystrophin protein leads to increased membrane fragility, myocyte death, fibrosis, and progressive loss of muscle strength. While more is known regarding the genetic perturbations underlying DMD, available treatments to counteract or prevent debilitating muscle weakness are still limited and lack efficacy for this and other muscular dystrophies (Rahimov and Kunkel, 2013).

### Centronuclear muscle myopathies

Centronuclear myopathies (CNMs) are inherited neuromuscular disorders with features of congenital myopathy that are characterized by a high proportion of myofibers with centrally located nuclei (Wallgren-Pettersson et al., 1995; Pierson et al., 2005; Tosch et al., 2006). Normally, the nucleus is found along the edges of rod-shaped muscle cells but in people with CNM the nucleus is located in the center of these cells. It is unclear exactly how the change in the location of the nucleus directly affects muscle cells. It is likely that absence of the cellular mechanism responsible for moving nuclei to the periphery, which could involve defects in the cytoskeleton, is involved in the etiology of this myopathy (Jungbluth et al., 2008). Centronuclear myopathies are divided into forms based on their proposed pattern of inheritance, associated symptoms and muscle pathology. Mutations to the dynamin 2 gene (*DNM2*) were recently associated with the autosomal dominant form of CNM while X-linked CNMs were associated with mutations in *DNM2*, *AMPH* (amphiphysin), *MTM1* (myotubularin) and most recently *MTMR14* (myotubularin-related protein-14) genes (Laporte et al., 1996, 2003; Jungbluth et al., 2008; Romero, 2010; Romero and Bitoun, 2011). How mutations to these genes lead to the observed muscle weakness and other specific features of these myopathies is unclear but it is suggested that intracellular trafficking of essential molecules is disrupted. The functional impact of the observed progressive muscle weakness in CNMs is much greater than would be expected if based simply on the loss of muscle mass leading to interest in exploring other aspects of muscle physiology including muscle strength and fatigue.

### A look at key genetic perturbations underlying muscle wasting disorders

Many genes have been proposed to be associated with various muscle wasting disorders but the mechanisms by which these perturbations cause the observed pathology is complex and remains unclear (Sewry, 2008). These proposed genes are involved in a multitude of biochemical processes that are essential for proper functioning of skeletal muscles including but not limited to stabilizing cell membranes, Ca^2+^ handling required for proper contraction and relaxation of muscles, and proper ubiquitin-mediated degradation of proteins (Teixeira Vde et al., 2012). While the loss of muscle mass is a critical part of these disorders, ultimately it is the progressive muscle weakness that increases the morbidity and mortality associated with musculoskeletal disorders. By understanding the biochemical processes leading to the observed phenotypes, treatments and therapeutics can be designed to reduce the functional impact of muscle weakness and slow its progression.

**Figure 1:**
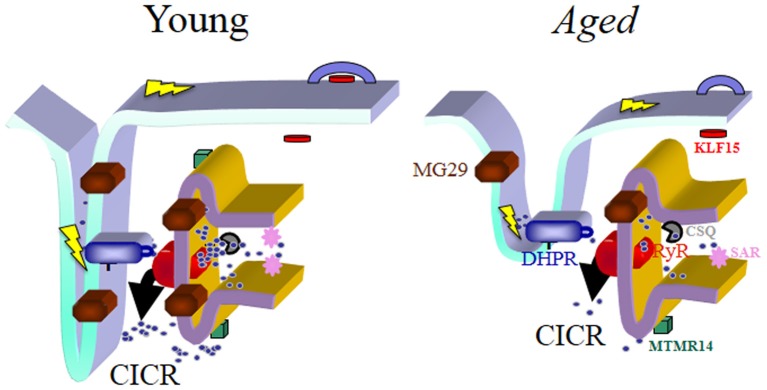
**Schematic drawing of the triad junction, the chief site of the E-C coupling process in skeletal muscles.** The predicted localization of the four genes/proteins emphasized in this review article is shown and they are represented in different colors along with the Dihydropyridine Receptor (DHPR), the Ryanodine Receptor type 1 (RyR1) and Calsequestrin (CSQ). In young muscles E-C coupling is effectively maintained through coordinated actions of the E-C coupling machinery and the optimal participation of MG29, MTMR14, SAR, and KLF15. Their concentration and/or effectiveness is reduced with aging, which associates with structural changes of the triad junction itself. Together these biochemical and morphological changes contribute to the reduced coupling between depolarization of the sarcolemma and contraction due to the reduced calcium release capacity of aged muscles. In summary, “E-C coupling quality” is reduced in aged muscles, and becomes a key factor to reduced muscle quality during aging. The steps of the E-C coupling process are described in detail in the text. In skeletal muscles, depolarization of the sarcolemma and its invaginations (t-tubules) represented by the lightning bolt in yellow color alters the configuration of the DHPR, which modifies its interaction with RyR1, leading to the dominant type of calcium release in skeletal muscle (depolarization-induced calcium release, DICR). This initial release phase can be further amplified by a secondary mechanism, calcium-induced calcium release (CICR), the main release mechanism in cardiac muscles. The structural deformation as well as the lack of organized triads is a hallmark of aged muscles and also common in other diseases covered in this article. Not detailed in this figure is the process of calcium entry or re-entry, store-operated calcium entry (SOCE), responsible for continual refilling of the sarcoplasmic reticulum (SR). SOCE is also reduced with aging, which we have postulated contributes to sarcopenia and to the un-matching between muscle mass and muscle contractile force during aging, since force/power decrease significantly more than the observed decrease in muscle mass. We foresee that new generations of drugs could be developed to specifically target the different steps of E-C coupling in disease states to increase efficiency of Ca^2+^ handling.

Skeletal muscle serves many functions throughout the human body beyond controlling muscle contraction including the more recent focus on its role in body metabolism and in metabolic and physical inactivity disorders. In this section, the unique key functions of specific genes, including *MTMR14*, *SAR* (sarcalumenin), *MG29*, and *KLF15*, will be discussed. Over the past few years, these genes have provided novel insights about muscle function in health and disease including muscle fatigue, muscle metabolism, and muscle aging (see Figure 1). These are major health issues as indicated by the large global impact of musculoskeletal disorders on both morbidity and mortality.

## MTMR14 plays a key role in calcium homeostasis and regulation of autophagy in skeletal muscle cells

Myotubularin (MTM) and myotubularin-related (MTMR) genes belong to a family of genes that encode dual-specificity phosphatases that modify phosphoinositides and regulate membrane traffic (Dowling et al., 2009, 2010). Genes in this family are shown to be important in muscle cell differentiation and mutations to *MTM* and *MTMR* genes are observed in patients with specific centronuclear myopathies (Laporte et al., 1996, 2003; Wishart and Dixon, 2002; Dowling et al., 2009, 2010). In 2010, multiple research groups identified mutations to *MTMR14*, one member of this gene family, in several patients with CNMs and in sarcopenia (Tosch et al., 2006; Dowling et al., 2009; Romero-Suarez et al., 2010). The MTMR14 gene was first identified as a cytoplasmic localized phosphatase specific to skeletal and cardiac muscle (Tosch et al., 2006; Vergne et al., 2009). Initially, this gene was named as muscle-specific inositol phosphatase (MIP) or Jumpy but when this gene was found to have high homology to the catalytic motif of myotubularin family proteins, it was renamed MTMR14 (Alonso et al., 2004; Tosch et al., 2006; Shen et al., 2009). MTMR14 specifically targets 1-O-(3-sn-phosphatidyl)-1D-myo-inositol 3-(dihydrogen phosphate) (phosphatidylinositol 3-phosphate (PtdIns(3)P)) and L-alpha-phosphatidyl-D-myo-inositol 3,5 bisphosphate, dipalmitoyl (phosphatidylinositol 3,5-diphosphate (PtdIns(3,5)P) substrates (Tosch et al., 2006; Dowling et al., 2009; Gibbs et al., 2010; Romero-Suarez et al., 2010). The products of this reaction are PtdIns and PtdIns(5)P, which are involved in cytoskeletal dynamics and intracellular membrane trafficking.

Using *MTMR14* specific knockout mice, a number of research groups explored the function of *MTMR14* and identified MTMR14 as a contributory factor in sarcopenia. Initial studies in these mice found excess PtdIns(3,5)P in skeletal muscle cells confirming this as a substrate of MTMR14 and suggesting this substrate plays an active role downstream (Shen et al., 2009; Romero-Suarez et al., 2010). Altered abundance of this phosphoinositide substrate and other phosphoinositides can cause defects in Ca^2+^ homeostasis thus creating a possible link for MTMR14 function in skeletal muscle pathophysiology.

Brotto and collaborators observed young *MTMR14* knockout (KO) mice exhibited impaired Ca^2+^ homeostasis, decreased muscle contractile force, and loss of muscle mass, all of which are reminiscent of aging (Figure 1). In comparing muscles of young and old wild type mice, levels of MTMR14 protein were reduced in older wild type mice (Romero-Suarez et al., 2010). Similarly, Shen et al. found *mtmr14*^−/−^ mice were prone to greater skeletal muscle fatigue and these mice showed decreased motor function including decreased walking speed and decreased running time prior to exhaustion. Extensor digitorum longus (EDL) muscles of these knockout mice had a 60% decrease in force-generating capacity and a prolonged relaxation profile post-muscle contraction compared to wild type mice of the same age. When the fatigue-resistant, slow-twitch soleus muscles of these KO mice were examined, they showed a shift toward higher frequencies while exhibiting greater fatigue and diminished recovery post-fatigue (Shen et al., 2009). This shift may reflect lesser Ca^2+^ release from the SR, therefore at higher frequencies of stimulation more calcium can be released. These defects could be explained by the alterations in calcium homeostasis, particularly the reduced availability of calcium for effective calcium release during contraction/relaxation cycles.

Knockout mice also exhibited defects in the regulation of Ca^2+^ levels including elevated resting Ca^2+^ concentrations, decreased Ca^2+^ content in the sarcoplasmic reticulum (SR), and prolonged release or defective Ca^2+^ clearance in these muscles. While store-operated calcium entry (SOCE) was functional in these mutant muscles, it was severely blunted and associated with muscle weakness and impairment of muscle relaxation. The findings from *MTMR14* knockout mice point to a role of the MTMR14 phosphatase in regulating Ca^2+^ essential for excitation-contraction coupling and SOCE function. Defects in these processes result in muscle fibers that are more susceptible to exercise-induced muscle damage and will lead to muscle weakness, both of which are trademarks of muscle wasting especially in sarcopenia (Zhao et al., 2008; Romero-Suarez et al., 2010) (Figure 1). For example, elevated levels of Ca^2+^ can lead to activation of proteolytic enzymes and dysfunctional autophagy. Dowling et al. (2010) determined that while MTMR14 is required for motor function, it is not essential for myocyte homeostasis or normal embryonic development. Morpholino-mediated knockdown of MTMR14 in zebrafish resulted in morphological abnormalities and a developmental motor phenotype characterized by diminished spontaneous contractions and impaired excitation-contraction coupling. Unlike knockdown of another member of this gene family, MTM1, in this model, knockdown of *MTMR14* did not affect muscle ultrastructure (Shen et al., 2009). MTMR14 appeared to act in concert with MTM1 in the development of muscle pathology since simultaneous knockdown of both genes impaired motor function and muscle ultrastructure (Dowling et al., 2009). The resulting phenotype was more severe than that observed with knockdown of either gene alone. Analysis following the knockdown of both of these genes suggested the phenotype observed is likely mediated by an increase in autophagy (Dowling et al., 2010). Defects in Ca^2+^ homeostasis observed in *mtmr14*^−/−^ mice may be an initiator of this observed autophagy.

The role of MTMR14 as a negative regulator of autophagy was further evaluated by Gibbs et al. who observed several mutations in the *MTMR14* gene in cases of CNM (Gibbs et al., 2010). In these studies, knockdown of MTMR14 *in vitro* and *in vivo* significantly changed muscle function especially in embryos where decreased developmental motor activity and pronounced fatigability were observed. Knockdown also increased basal and starvation-induced autophagy of muscle cells demonstrated by increased LC3-II levels (Vergne et al., 2009; Dowling et al., 2010). During autophagy, the cytoplasmic form of LC3 (LC3-I) is processed to create LC3-II, which is recruited to autophagosomes. This conversion is used to monitor autophagic activity and LC3-II is considered an autophagic marker. A possibility that MTMR14 may act as a modifier of disease rather than direct cause has been raised. While function-altering MTMR14 mutations have been found in sporadic cases of centronuclear myopathy, one of these patients also carried a disease-associated mutation in *DNM2*. A more severe phenotype was observed in this patient than patients with only the *DNM2* mutation suggesting MTMR14 was not the primary cause of disease but did have a role in exacerbating the phenotype (Bitoun et al., 2005; Dowling et al., 2010).

The mutation of *MTMR14* in cases of CNM and the decreased presence of MTMR14 protein in muscles from aged mice suggest the importance of MTMR14 in muscle physiology and pathophysiology. MTMR14 studies have identified its roles in regulating the abundance of phosphatidylinositol phosphates, which observably alters Ca^2+^ homeostasis. Changes in Ca^2+^ concentrations are a known inducer of autophagy supporting the increased levels of autophagy observed following MTMR14 knockdown (Bonaldo and Sandri, 2013; Smaili et al., 2013). Functionally, MTMR14 affected muscle performance specifically muscle fatigue and muscle weakness.

One key message from these MTMR14 studies is that finely controlled levels of phosphoinositides in muscle cells is essential for maintaining Ca^2+^ homeostasis and enabling effective muscle performance. Improper regulation of Ca^2+^ concentrations by any means, including that observed with these MTMR14 studies, interferes with excitation-contraction (E-C) coupling, which is another physiological process fundamental to muscle pathology. E-C coupling, the conversion of an electrical stimulus by cells to a mechanical response, is aberrantly regulated in various pathologies including muscle wasting disorders (Yoshida et al., 2005; Rossi and Dirksen, 2006). In skeletal muscle, E-C coupling requires two specific proteins, the sarcoplasmic reticulum Ca^2+^ release channel (known as the ryandonine receptor or RyR) and voltage-gated Ca^2+^ channels (known as dihydropyridine receptors or DHPRs). Depolarization of the membrane potential of these cells by an action potential activates voltage-gated DHPRs. This activates RyR type 1 via physical linkage and conformational changes. As RyRs open, Ca^2+^ is released from the SR into the junctional space then diffuses into the cytoplasm to cause a Ca^2+^ transient (Bellinger et al., 2008; Andersson et al., 2011). Ca^2+^ released into the cytoplasm binds to Troponin C on actin filaments to produce force or contraction of the cell. The sarco/endoplasmic reticulum Ca^2+^ ATPase (SERCA) pumps Ca^2+^ back into the SR and with this the force begins to decline and relaxation occurs. The SR is the dynamic Ca^2+^ governor in muscle cells that receives feedback allowing it to maintain SR and cytoplasmic Ca^2+^ levels. The SR contains an elaborate set of Ca^2+^ regulating proteins including luminal Ca^2+^ binding proteins involved in Ca^2+^ storage, SR Ca^2+^ release channels, and SERCA pumps for Ca^2+^ reuptake. Spatial and temporal control of Ca^2+^ uptake, Ca^2+^ buffering, and Ca^2+^ release is maintained by these highly organized Ca^2+^ regulatory proteins in the SR (O'Connell et al., 2008) (Figure 1).

## Sarcalumenin functions in calcium handling

Another gene related to E-C coupling with a role in muscle pathology is sarcalumenin (*SAR*). Similar to calsequestrin, sarcalumenin is a Ca^2+^ binding protein localized to the sarcoplasmic reticulum of the intracellular Ca^2+^ store of striated muscle cells. While calsequestrin and sarcalumenin are both Ca^2+^ binding proteins of the SR, they are located in different regions of the SR. Sarcalumenin observably colocalizes with SERCA. Two isoforms of SAR, a 160 kDa and a 35 kDa glycoprotein are formed as the products of alternative splicing of the primary transcript (Leberer et al., 1989, 1990). The SAR luminal protein binds Ca^2+^ with high capacity but low affinity (Zhao et al., 2005). Increased SAR expression during muscle development suggests its role in proper functioning of mature SR (Yoshida et al., 2005). Currently, sarcalumenin is considered to be important in the release and uptake of Ca^2+^, which is the essential second messenger of the excitation-contraction-relaxation cycle in skeletal muscle cells (Yoshida et al., 2005; Rossi and Dirksen, 2006; O'Connell et al., 2008). Using a *SAR* knockout mouse, Yoshida et al. determined that while SAR is not essential for fundamental muscle function, it does play a role in improving Ca^2+^ handling functions of the SR in striated muscle. Muscle from *sar*^−/−^ mice exhibited weakened Ca^2+^ uptake in isolated SR vesicles. In mutant muscles, expression of SERCA protein was decreased while levels of the mRNA remained consistent with wild type muscles. Sarcalumenin unlike most proteins of the SR lacks the four amino acid (KDEL) ER/SR retention signal so it is likely that the direct interaction of SAR with SERCA serves this function (Leberer et al., 1990; Yoshida et al., 2005; Dowling et al., 2009). This is suggestive of SAR acting as a chaperone of SERCA and its involvement in SERCA turnover since the absence of SAR directly impacts the abundance of SERCA protein. Together these findings suggest SAR contributes to Ca^2+^ buffering and the maintenance of Ca^2+^ pump proteins, both of which are essential for E-C coupling and retaining muscle strength. From current research, it remains inconclusive whether SERCA content or a combination of SERCA content and buffering capacity is responsible for the observed effects linked to sarcalumenin expression.

Reduced levels of SAR protein were detected in the dystrophin deficient *mdx* mouse model of muscular dystrophy leading Zhao et al. to further evaluate its role in muscle wasting (Dowling et al., 2004; Zhao et al., 2005). The *sar*^−/−^ mouse model exhibited enhanced fatigue resistance. This finding was determined by evaluating the Ca^2+^ ion storage function of the contractile machinery using single, mechanically skinned muscle fibers loaded with two calcium dyes, one that specifically reported t-tubule calcium and the other that reported SR calcium (Zhao et al., 2005). A number of key findings were obtained using this method for comparison of wild type and *sar*^−/−^ mice. Muscle fibers from SAR deficient mice showed elevated SOCE activity as previously observed and reduced fatigability. Putting the findings from these studies together suggests the fatigue resistant phenotype of *sar*^−/−^ mice is likely due to more effective E-C coupling and SOCE observed in these muscles.

While ATP is thought to play a major role in fatigue, it is typically associated with long-term fatigue under specific conditions. It has been demonstrated by many laboratories that a major culprit in the muscle fatigue process is dysfunctional intracellular calcium homeostasis, specifically impaired Ca^2+^ release from the SR. In normal muscles, calcium stores are more easily depleted than ATP stores and normal muscle function is directly related to Ca^2+^ availability. As less Ca^2+^ becomes available with each contraction/relaxation cycle, fatigue will develop if SOCE is reduced (Brotto et al., 2002; Weisleder et al., 2006; Allen et al., 2008; Place et al., 2009). The studies discussed here concluded the role of sarcalumenin in SOCE function may be useful in reducing or reversing weakness associated with various muscle wasting disorders. Additionally, these studies raised the possibility that the reduced levels of SAR protein observed in *mdx* mice could be interpreted as a compensatory mechanism of adaptation in muscles from these mice in an effort to improve muscle function (Zhao et al., 2005). Further studies suggested that another gene Mitsugumin-29 (*MG29*), might be involved with the compensation observed in the *sar*^−/−^ muscle.

## MG29 functions in muscle fatigue and store-operated calcium entry

MG29 is another protein of interest for its identified role in muscle physiology and pathology. Over the last ten years, research has evaluated the role of MG29 in muscle fatigue and SOCE. Using an extensive proteoimmunologic library of antibodies that targeted proteins of the triad junctional membrane structures of skeletal muscle, MG29 was one of the most significant proteins identified (Takeshima et al., 1998). Mitsugumin-29 (MG29) is a member of the synaptophysin family of transmembrane proteins that has been extensively evaluated for their role in muscles. MG29 is almost exclusively expressed in skeletal muscle fibers (Takeshima et al., 1998). MG29 contains four transmembrane domains with a cytoplasmic amino and carboxy terminus. The transmembrane domains allow MG29 to localize at both the transverse (t-) tubular membrane and the SR membrane of the triad junction, which suggests a possible role of MG29 in mediating communication between t-tubular and junctional SR membranes (Thomas et al., 1988, 1998; Thomas and Betz, 1990).

Besides the homologous amino acid sequence, MG29 also shares other characteristic structural features with members of the synaptophysin family of neurotransmitters. Synaptophysin was identified as an abundant immunogenic membrane protein of small synaptic vesicles and is also found in neurosecretory granules (Thomas et al., 1988, 1998; Thomas and Betz, 1990). The structural role of synaptophysin in synaptic vesicle biogenesis and its tight interaction with other proteins of the synaptic vesicle membrane contribute to its essential role in neurotransmitter secretion (Thiele et al., 2000). Similarities between the structure and localization patterns of MG29 to synaptophysin suggest MG29 has an important role in modulating membrane structures in skeletal muscle. Skeletal muscle is one of the most plastic tissues in the human body and since normal muscle physiology requires the formation and maintenance of complex membrane structures, it has been proposed that MG29 may be the structural counterpart of synaptophysin in skeletal muscle biogenesis and maintenance (Booth et al., 2000; Booth and Vyas, 2001).

Mutations of *MG29* have not been observed in specific skeletal muscle wasting disorders; however, its expression and abundance are observed to vary under certain conditions. For example, MG29 expression is known to decrease in aging mouse skeletal muscle (Weisleder et al., 2006). To determine the physiological role of MG29 in normal muscle function and possibly in muscle pathology, an *MG29* knockout mouse was established. The MG29 null mouse was the first experimental indication of the role MG29 plays in muscle membrane integrity. Skeletal muscle from these mice showed multiple abnormalities in membrane structure specifically around the triad junction. Within the triad junction, t-tubules appeared swollen and the SR networks were poorly formed with fragmented structures (Nishi et al., 1999; Thiele et al., 2000). Based on what is known regarding synaptophysin, these findings suggest MG29 functions in membrane fusion associated with the creation and maintenance of membrane structures in the triad junction.

Considering the extent of malformation of the triad junction membrane ultrastructure, the lack of a functional impact of *MG29* knockout was surprising so the phenotype was further evaluated under conditions of physiological stress (Nagaraj et al., 2000). During treadmill running, MG29 knockout mice ran significantly less and were unable to sustain physical activity for the extended period of time compared to littermate controls suggesting a direct role of MG29 in muscle performance specifically during increased physical activity. Additional *ex vivo* muscle contractility assays confirmed increased fatigability in isolated *mg29^−/−^* muscles. *MG29* null muscles fatigued to a greater extent while also recovering less after fatigue. These muscles also produced less force than wild type control mice even with the addition of caffeine. These findings continue to suggest that E-C coupling in MG29 ablated skeletal muscles is disrupted since muscle fatigue was reduced in *mg29^−/−^* muscles when Ca^2+^ was removed from the extracellular medium and by pharmacologically blocking extracellular Ca^2+^ entry (Nagaraj et al., 2000). It is clear that the inability of humans to sustain physical activity may lead to chronic degenerative diseases, reduced muscle function, and muscle wasting (Booth et al., 2000). However, the contribution of changes in fatigability to sarcopenia and age related frailty has not been fully resolved as some reports indicate some degree of fatigue resistance develops during muscle aging (Gonzalez and Delbono, 2001a,b). Given that MG29 levels decrease in aging skeletal muscle these findings show that muscle aging is a multivariate situation where changes in multiple factors contribute to the development of aging phenotypes, and emphasize the need for additional studies in this important area of investigation (Weisleder et al., 2006).

The implication of extracellular Ca^2+^ entry as a major factor in muscle fatigue in *mg29^−/−^* muscle lead to investigation of whether store-operated calcium entry (SOCE) is altered in these muscles compared to wild type control muscles. SOCE is an extracellular calcium entry pathway. In SOCE, reduced Ca^2+^ concentration in the intracellular stores of the sarcoplasmic reticulum induces influx of Ca^2+^ from the extracellular space to replenish the diminished Ca^2+^ stores. While SOCE functions in many different cell types, it is extremely important in the physiology of excitable cells such as muscles and neurons (Albert and Large, 2003; Ma and Pan, 2003; Nilius, 2004; Targos et al., 2005; Ma et al., 2006; Lewis, 2007). Disruption of SOCE activity results in various physiological pathologies (Nilius, 2004; Targos et al., 2005). Its impairment can lead to numerous disorders including cancer and primary immunodeficiency and is being continually researched for its possible role in Alzheimer disease and age-related muscle weakness (sarcopenia) (Nilius, 2004; Targos et al., 2005; Lewis, 2007). The diverse pathologies linked to SOCE is likely due to the wide ranging importance of Ca^2+^ as a second messenger in controlling cellular functions including contraction, secretion, gene expression, and cell cycle.

SOCE is important in long term maintenance of Ca^2+^ homeostasis since it is the mechanism by which additional Ca^2+^ is provided for muscle contraction under conditions where SR Ca^2+^ is depleted such as fatigue, intense exercise, and some pathologies (Zhu and Birnbaumer, 1998; Elliott, 2001; Putney et al., 2001; Parekh and Putney, 2005; Yoshida et al., 2005; Zhao et al., 2005, 2008). Elevated SOCE can also be detrimental as increased resting intracellular Ca^2+^ concentrations potentially underlies development and progression of the muscle injury observed with muscular dystrophy (Brotto et al., 2002). Together, these differential effects of aberrant SOCE suggest its fine-tuned modulation is essential for overall skeletal muscle health. Reduced SOCE was displayed in *MG29* null muscles supporting a role of Ca^2+^ entry in the observed phenotypic changes (Pan et al., 2002). *mg29^−/−^* muscles fatigued to a greater extent when blockers of SOCE were employed which suggests that the main problem in Ca^2+^ handling is due to reduced SOCE leading to reduced SR calcium storage. Additional studies using more specific SOCE antagonists and genetically silencing players in SOCE will shed more light on this mechanism. Aberrant reduction of SOCE was mirrored in aged skeletal muscles, which also demonstrated decreased expression of MG29 protein resulting in a direct correlation of MG29 protein levels and fatiguing of skeletal muscles.

Additional evidence to support the role of MG29 in SOCE and muscle fatigue stems from the sarcalumenin knockout mouse previously discussed (Yoshida et al., 2005). Muscles isolated from *sar*^−/−^ mice exhibited reduced fatigability and elevated SOCE activity compared to wild type mice. These observed features correlated with increased abundance of MG29 (Zhao et al., 2005). Based on the increased susceptibility of MG29 null muscles to fatigue, it is proposed that the increased presence of MG29 in *sar*^−/−^ mice may be compensatory for the loss of SAR. The compensatory mechanism would contribute to enhanced Ca^2+^ release from the SR and more efficient SR coupling in these muscles. This finding further supports the role of both MG29 and sarcalumenin in maintenance of Ca^2+^ homeostasis and suggests both genes as targets for restoring muscle strength. Putting all these findings together, MG29 may function as a sentinel against age related dysfunction in skeletal muscle by its likely role in regulation of SOCE. MG29 and SAR may serve as therapeutic targets for pathophysiologic muscle conditions including aging and dystrophy where muscle fatigue and strength are impacted. The two main physiologic effects on skeletal muscle by muscle wasting disorders are the physical loss of muscle mass and increased muscle weakness. Both are important processes in the effort to maintain proper functioning of skeletal muscles especially with age. The genes covered above, *MTMR14*, *SAR*, and *MG29*, are being heavily researched for their role in muscle weakness and the induction of fatigue in these disorders. On the other hand, KLF15 has been evaluated for its role in the loss of muscle mass and its possible role in muscle weakness.

## Kruppel-like factor 15

*Kruppel-Like Factor 15* (*KLF15*) belongs to the Kruppel-like factor (KLF) family of transcription factors in a zinc-finger class of DNA binding transcriptional factors. Their transcriptional activity makes their function critical in muscle physiology and muscle pathophysiology. KLFs include three Cys_2_/His_2_ containing zinc fingers, all of which are located at the extreme c-terminus of the protein. The seven residues separating these zinc fingers are also highly conserved but the non-DNA binding regions of these factors are highly divergent allowing modulation of transactivation or transrepression and mediating protein-protein interactions (Bieker, 1996; Turner and Crossley, 1999; Haldar et al., 2007, 2012; Pearson et al., 2008) This sequence variation likely contributes to the varied expression patterns and functions of the family members. KLFs are differentially expressed in development as well as in response to physiological stresses and are found to play critical roles in cardiovascular biology and in muscle biology, including skeletal muscle (Yamamoto et al., 2004; Haldar et al., 2007). Of specific interest in skeletal muscle biology is KLF15. KLF15 is expressed in all three types of muscle and is known to be a negative regulator of hypertrophic remodeling within the heart by repressing key features required for the hypertrophic process (Fisch et al., 2007; Haldar et al., 2007, 2012).

Research over the past ten years has discovered the importance of KLF15 in skeletal muscle metabolism for both amino acid catabolism and lipid utilization. Its role in metabolism may contribute to its association with muscle atrophy. In two separate studies, KLF15 was evaluated for its function in muscle atrophy and hypertrophy. Schakman et al. researched KLF15 expression in response to glucocorticoids (GC). Long term administration of glucocorticoids as treatment for specific diseases results in debilitating muscle atrophy (Schakman et al., 2013). Biochemically, glucocorticoids were found to increase the rate of protein breakdown and decrease the rate of protein synthesis thus contributing to a loss of muscle mass (Tomas et al., 1979; Goldberg et al., 1980; Lofberg et al., 2002; Drummond and Rasmussen, 2008; Schakman et al., 2013). It was determined that mTORC1 signaling is repressed in the presence of increased GCs through enhanced transcription of *REDD1* and *KLF15*. The mechanism by which KLF15 contributes to protein catabolism is not completely understood but KLF15 appears to activate branched-chain amino acid aminotransferases (BCAT) that are responsible for the degradation of branched chain amino acids (BCAAs). Accelerated BCAA degradation leads to the observed decrease in mTORC1 activity (Chaillou et al., 1985; Bodine et al., 2001; Shimizu et al., 2011; Atherton and Smith, 2012). Further analysis showed that KLF15 in coordination with FOXO1 upregulates E3 ubiquitin ligases Atrogin-1 and MURF1 (Schakman et al., 2013). KLF15 appears to contribute to muscle atrophy by altering the ratio of protein catabolism and synthesis.

While studying the development of muscle hypertrophy, Chaillou et al. recently discovered that KLF15 expression is downregulated in response to overload induced hypertrophy. Decreased KLF15 expression in skeletal muscle resulted in greater protein accretion possibly through decreased degradation of BCAAs leading to increased availability of BCAAs and maintained synthesis of proteins (Chaillou et al., 1985). The mechanism by which KLF15 affects protein synthesis through altered BCAA degradation remains unclear due to the complex nature of this process. Protein synthesis depends on specific roles of the liver and constant crosstalk between the liver and skeletal muscles as well as the commonly ignored function of insulin, which also stimulates skeletal muscle utilization of amino acids/proteins, besides its more readily, attributed effect on glucose uptake. In a parallel study, increased concentrations of BCAAs prolonged activation of mTORC1, which is vital to the process of muscle hypertrophy by enhancing protein synthesis. For this reason, KLF15 may be a target of future studies for controlling skeletal muscle hypertrophy and more specifically skeletal muscle atrophy associated with certain conditions. It appears repression of KLF15 may beneficially impact skeletal muscle by restoring muscle cell loss.

While the role of KLF15 in protein synthesis and catabolism has been researched, KLF15 has also been shown to have a role in lipid utilization in skeletal muscles. With its regulation of amino acid catabolism and its role in fasting gluconeogenesis, KLF15 is proposed to have a role in metabolic adaptation in conditions of physiologic stress such as endurance exercise and fasting (Gray et al., 2007; Shimizu et al., 2011; Haldar et al., 2012). In the lack of sufficient glucose, which is vital for brain function, skeletal muscle resorts to proteins and lipids for its fuel. Specifically, abnormalities of lipid flux impact energetic failure and tolerance to exercise. Studies by Haldar et al. propose that KLF15 and its signaling interaction with glucocorticoids are important in the physiological response to exercise based on its role in both protein and lipid metabolism (Haldar et al., 2012). Yamamoto et al. observed that fasting elevated levels of KLF15 and this lead to increased expression of the mitochondrial acetyl-CoA synthetase gene AceCS2 in skeletal muscle (Fujino et al., 2001; Yamamoto et al., 2004). This enzyme is vital during periods of insufficient glucose and under ketogenic conditions for providing an energy source for muscles (Yamamoto et al., 2004; Gray et al., 2007). The exact role of KLF15 in metabolic lipid flux in skeletal muscle remains unknown but research is focused on understanding the role of KLF15 in defective lipid flux and protein catabolism, both of which can contribute to detrimental changes in muscle cells. Atrophy can be a result of these defects as muscles try to adapt to metabolic changes. In addition to its evaluation in atrophy, KLF15 has been linked to facioscapulohumeral dystrophy (FSHD). For FSHD, KLF15 activated the D4Z4 enhancer element leading to overexpression of DUX4 while this effect was abolished with silencing of *KLF15*. DUX4 is known to be overexpressed in this type of muscular dystrophy (Dmitriev et al., 2011). KLF15 appears to be a promising research target in understanding how metabolism of proteins and lipids may contribute to muscle atrophy as well as its possible role in muscle wasting disorders such as types of muscular dystrophy (Chaillou et al., 1985).

## Conclusions and future directions

The available research on MTMR14, sarcalumenin, MG29, and KLF15 in skeletal muscle biology has provided new information toward the better understanding of muscle physiology and pathophysiology. Specific interest is focused on these genes and their possible roles in muscle weakness including that observed during the natural aging process (Figure 1). In aging, the most debilitating effects of sarcopenia are the increased muscle weakness and fatigue. This is the case for many other muscle wasting disorders. Muscle wasting disorders display a loss of muscle mass but of greater functional importance is the increased muscle weakness. Muscle weakness is the pathophysiological condition that has the greatest effect on quality of life, independence, and outcome for individuals affected by these disorders. The genes reviewed here are observed to affect key processes in muscle function including SOCE and E-C coupling, which both have proposed roles in muscle fatigue and weakness. While the underlying mechanisms behind these genes and other genes in the loss of muscle strength are finally coming to light, hope is that with additional research, these genes will provide new avenues in finding a therapeutic target in the fight against muscle wasting disorders. We foresee that a new generation of drugs that specifically modulate E-C coupling and calcium homeostasis would be great assets in this fight that is currently being won by the diseases.

## Author contributions

Dr. Manring drafted and organized the entire review. Drs. Weisleder and Brotto have had substantial contributions to the conception or design of the work, the acquisition, analysis, and interpretation of data on the genes presented in this review. Dr. Leticia Brotto has contributed during more than one decade in conducting a large amount of the many experiments that led to characterization of the knockout animal models discussed in this article. Dr. Abreu critically revised the initial draft for intellectual and organization content. Dr. Brotto conducted the final approval of the version to be published in agreement with all the authors and he is accountable for all aspects of the work in ensuring that questions related to the accuracy or integrity of any part of the work are appropriately investigated and resolved.

### Conflict of interest statement

Dr. Noah Weisleder is the Founder and Chief Scientific Officer of TRIM-edicine, a biotechnology company developing protein therapeutic agents. The other authors declare that the research was conducted in the absence of any commercial or financial relationships that could be construed as a potential conflict of interest.
